# Design of a Compact Transfer Radiometer for a Radiometric Benchmark Transfer Chain

**DOI:** 10.3390/s22186795

**Published:** 2022-09-08

**Authors:** Kaichao Lei, Xin Ye, Zhiwei Xia, Nan Xu, Shuqi Li, Yachao Zhang, Yuwei Wang, Zhiwei Liu, Zhigang Li

**Affiliations:** 1 Changchun Institute of Optics, Fine Mechanics and Physics, Chinese Academy of Sciences, Changchun 130033, China; 2 School of Optoelectronics, University of Chinese Academy of Sciences, Beijing 100049, China; 3 National Institute of Metrology, Beijing 100029, China

**Keywords:** transfer radiometer, cryogenic radiometer, radiance, calibration

## Abstract

In order to meet the high-accuracy calibration requirements of satellite remote sensing instruments, a transfer radiometer for an on-orbit radiometric benchmark transfer chain has been developed, which provides vital technical support for realizing the radiometric calibration uncertainty of the order of 10^−3^ for remote sensing instruments. The primary role of the transfer radiometer is to convert from the spectral power responsivity traceable to a cryogenic radiometer to the spectral radiance responsivity and transfer it to the imaging spectrometer. At a wavelength of 852.1 nm, the combined uncertainty of the radiance measurement comparison experiment between the transfer radiometer and a radiance meter is 0.43% (k = 1), and the relative deviation of the measurements between the transfer radiometer and the radiance meter is better than 0.36%, which is better than the combined uncertainty of the radiance measurement comparison experiment. This demonstrates that the transfer radiometer can achieve radiance measurements with a measurement uncertainty better than 0.3% (k = 1).

## 1. Introduction

Remote sensing is essential for people to extract miscellaneous information related to the Earth’s environment. Accurate climate prediction is a considerable challenge facing the scientific community today. Space remote sensing technology is critical to improving our understanding of the Earth’s climate and the ability to accurately predict future climate change, which makes the demand for high-accuracy radiometric calibration of space on-orbit remote sensing instruments increasingly urgent. Even though pre-launch radiometric calibration of space remote sensing instruments can be accomplished with high accuracy in ground laboratories, a post-launch instrument may change due to various reasons. Therefore, the high absolute accuracy requirements from remote sensing data products still cannot be met if there is a lack of accurate on-orbit calibration, directly traceable to the International System of Units (SI) during on-orbit operation. Spacecraft missions, including Traceable Radiometry Underpinning Terrestrial- and Helio-Studies (TRUTHS) and Climate Absolute Radiance and Refractivity Observatory (CLARREO), that provide on-orbit SI traceable calibration have been proposed to enable high-accuracy space-based climate observing systems [1,2,3,4,5,6,7]. The Chinese Space-Based Radiometric Benchmark (CSRB) project has been under development since 2014, with the goal of launching the radiometric benchmark satellite using the new on-orbit calibration system [8]. The Space Cryogenic Absolute Radiometer (SCAR) is the on-board benchmark for achieving long-term radiometric measurements with high stability and high accuracy in the solar-reflective band. The intention of applying the primary standard SCAR is to calibrate the Earth-Moon Imaging Spectrometer (EMIS) on-satellite for spectral radiance responsivity by means of the benchmark transfer chain (BTC) and to transfer the traceable radiometric scale to other remote sensors via cross-calibration. The transfer radiometer plays a crucial role in the BTC. First, the transfer radiometer is calibrated directly against the SCAR for spectral power responsivity, using some laser diodes with discrete monochromatic wavelengths as light sources. The spectral power responsivity of the transfer radiometer can be converted into spectral radiance responsivity based on its own conversion coefficient from power responsivity to radiance responsivity. Then, the diffuser is observed simultaneously by the transfer radiometer and the imaging spectrometer. The transfer radiometer effectively measures the spectral radiance of the diffuser, which is then subsequently used as a calibrated quasi-Lambertian source to calibrate the imaging spectrometer. The radiometric scale of the SCAR is thus converted and transferred to the imaging spectrometer through the benchmark transfer chain (BTC).

Transfer radiometers have been widely used as calibration standards in radiometric measurement applications, such as for automated vicarious calibrations and evaluating the radiance and stability of sources used for optical sensor calibrations [9,10,11,12,13,14,15,16,17,18,19,20]. We developed a transfer radiometer for an on-orbit radiometric benchmark transfer chain, whose primary function is to convert from the spectral power responsivity traceable to a cryogenic radiometer to the spectral radiance responsivity and transfer it to the imaging spectrometer. The transfer radiometer radiance measurements are required to be traceable to SI, with an uncertainty of better than 0.3% (k = 1) to achieve the on-orbit high radiometric accuracy calibration of remote sensing instruments, such as imaging spectrometers. The SI-traceable transfer radiometer is suitable for radiant power, radiance, and irradiance measurements as a radiometric measuring instrument. The feature of the transfer radiometer, which can convert from power responsivity to radiance responsivity with high accuracy, is primarily adopted in the BTC. The transfer radiometer has a filter-free channel and 11 filter channels. The filter transmittance is affected by the angle of incidence of the ray, while the filter-free channel is not sensitive to the angle of incidence. Therefore, the power responsivity calibration is performed only for the filter-free channel of the transfer radiometer, and the conversion of power responsivity to radiance responsivity is completed based on this. Here we elaborate the design concept and structure of the transfer radiometer and depict the radiance measurement comparison experiment of the transfer radiometer and a radiance meter. In addition, we present the comparison results and evaluate the associated measurement uncertainties as well.

## 2. Design of Transfer Radiometer

The design of the transfer radiometer focuses on enabling the high-accuracy conversion of its spectral power responsivity to spectral radiance responsivity, since the spectral radiance responsivity of the imaging spectrometer is determined by comparison with the spectral radiance responsivity of the transfer radiometer. The transfer radiometer is mainly composed of a radiance measuring tube, a filter wheel, an integrating sphere, silicon, and extended InGaAs photodiode detectors. The transfer radiometer spectral power responsivity can be converted into spectral radiance responsivity by using two precision apertures [21].

**Figure 1:**
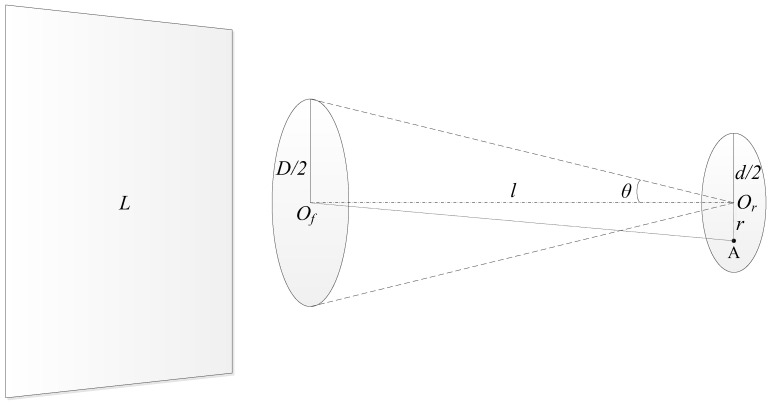
Schematic of radiance measurement.

As shown in Figure 1, the nominal viewing angle θ can be expressed as

(1) $$\theta =\arctan\frac{D}{2l}$$

where D is the diameter of the front aperture with the center point located at $O_{f}$, d is the diameter of the rear aperture with the center point located at $O_{r}$, l is the separation between the two apertures.

Assuming that there is a monochromatic Lambertian source with radiance L and a sufficiently large area prior to the front aperture, the irradiance $E_{r}$ at the center point $O_{r}$ of the rear aperture is [22]

(2) $$E_{r}=L\cdot \pi \sin^{2}\theta$$

Let the equivalent field of view (FOV) of the transfer radiometer be $\theta_{m}$, then the equivalent irradiance E on the entire rear aperture can be expressed as

(3) $$E=L\cdot \pi \sin^{2}\theta_{m}$$

Further, we get

(4) $$\theta_{m}=\arcsin\sqrt{\frac{E}{\pi L}}$$

Take any point A on the rear aperture and set ${\mathrm{AO}}_{r}=r$, then the irradiance $E_{A}$ at point *A* is [23]

(5) $$E_{A}=L\cdot \frac{\pi }{2}\left\{1-\frac{r^{2}+l^{2}-{\left(D/2\right)}^{2}}{{\left[r^{4}+2r^{2}(l^{2}-{\left(D/2\right)}^{2})+{\left(l^{2}+{\left(D/2\right)}^{2}\right)}^{2}\right]}^{1/2}}\right\}$$

When point A coincides with point $O_{r}$, there is

$$E_{A}=L\cdot \frac{\pi }{2}\left[1-\frac{l^{2}-{\left(D/2\right)}^{2}}{l^{2}+{\left(D/2\right)}^{2}}\right]=L\cdot \pi \frac{{\left(D/2\right)}^{2}}{l^{2}+{\left(D/2\right)}^{2}}=L\cdot \pi \sin^{2}\theta =E_{r}$$

When point A is inside the rear aperture, it can be considered that the irradiance of all points on the ring with point $O_{r}$ as the center and r as the radius is equal. The area of the rear aperture is $A_{d}=\pi {\left(d/2\right)}^{2}$, the equivalent irradiance E on the whole rear aperture is

(6) $$E=\frac{1}{A_{d}}\int_{0}^{d/2}E_{A}\cdot 2\pi rdr$$

As a result, there is

(7) $$\frac{E}{L}=\frac{1}{LA_{d}}\int_{0}^{d/2}E_{A}\cdot 2\pi rdr=\frac{\pi^{2}}{A_{d}}\int_{0}^{d/2}\left\{1-\frac{r^{2}+l^{2}-{\left(D/2\right)}^{2}}{{\left[r^{4}+2r^{2}(l^{2}-{\left(D/2\right)}^{2})+{\left(l^{2}+{\left(D/2\right)}^{2}\right)}^{2}\right]}^{1/2}}\right\}\cdot rdr$$

Substituting the above equation into Equation (4), the equivalent FOV $\theta_{m}$ of the transfer radiometer can be derived. The radiant power Φ passing the rear aperture in radiance measurement mode is

(8) $$\Phi =E\cdot A_{d}=L\cdot \pi \sin^{2}\theta_{m}\cdot A_{d}$$

For the transfer radiometer, the above equation gives the conversion relationship between the measured power Φ and the radiance L. Further, we can write a conversion equation between power responsivity and radiance responsivity of the transfer radiometer as

(9) $$R_{L}=R_{\Phi }\pi \sin^{2}\theta_{m}A_{d}=R_{\Phi }\frac{E}{L}A_{d}$$

where $R_{\Phi }$ is the power responsivity of the transfer radiometer, and $R_{L}$ is the radiance responsivity of the transfer radiometer. From Equations (7) and (9), the conversion relationship of transfer radiometer power responsivity to radiance responsivity can be expressed as

(10) $$R_{L}=R_{\Phi }C=R_{\Phi }\pi^{2}\int_{0}^{d/2}\left\{1-\frac{r^{2}+l^{2}-{\left(D/2\right)}^{2}}{{\left[r^{4}+2r^{2}(l^{2}-{\left(D/2\right)}^{2})+{\left(l^{2}+{\left(D/2\right)}^{2}\right)}^{2}\right]}^{1/2}}\right\}\cdot rdr$$

where $C=\pi^{2}\int_{0}^{d/2}\left\{1-\frac{r^{2}+l^{2}-{\left(D/2\right)}^{2}}{{\left[r^{4}+2r^{2}(l^{2}-{\left(D/2\right)}^{2})+{\left(l^{2}+{\left(D/2\right)}^{2}\right)}^{2}\right]}^{1/2}}\right\}\cdot rdr$ is an expression of the conversion coefficient C. This relationship shows that the conversion of power responsivity to radiance responsivity is affected by three parameters of the transfer radiometer radiance measuring tube: the diameter of the front aperture, the diameter of the rear aperture, and the separation between the two apertures. As long as the accurate values of the above three parameters are obtained, the high-accuracy conversion of power responsivity to radiance responsivity can be realized.

**Figure 2:**
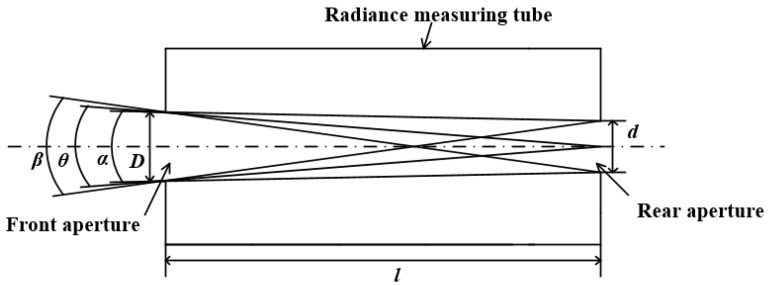
Schematic of FOV.

The front and rear apertures of the transfer radiometer are made of stainless steel, which can reduce the influence of thermal expansion and cold contraction and has the effect of corrosion resistance. Except for the front and rear apertures, all other parts of the radiance measuring tube are made of aluminum, minimizing the weight of the instrument. One of the transfer radiometer’s primary functions is calibrating the imaging spectrometer’s radiance responsivity. Therefore, the transfer radiometer should be designed to observe the same area as the imaging spectrometer as much as possible to reduce the radiometric calibration uncertainty. The diameters of the front and rear apertures of the transfer radiometer were measured using a universal tool microscope. The front aperture diameter D is 20.943 mm, and the rear aperture diameter d is 15.973 mm. The distance between the front aperture and the rear aperture was measured by a coordinate measuring machine. The separation l between the two apertures is 250.469 mm. Based on the above three parameters, four viewing angles of the transfer radiometer can be calculated. Full radiance-measurement angle, nominal viewing angle, and unvigenetted FOV are shown schematically in Figure 2. The viewing angle-specific parameters of the transfer radiometer are shown in Table 1.

**Figure 3:**
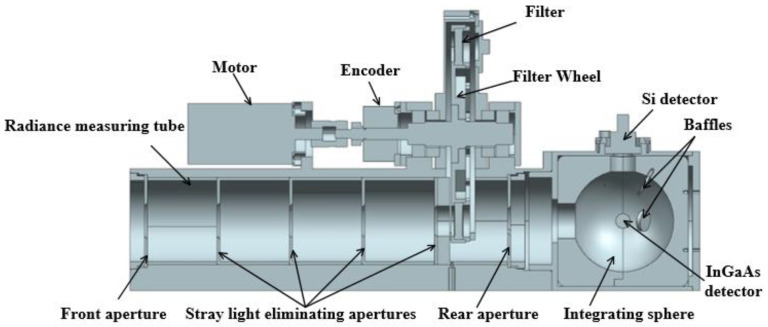
Sectional view of the transfer radiometer.

It will be necessary to minimize the effect of ambient stray light to achieve the high-accuracy conversion from power responsivity to radiance responsivity of the transfer radiometer. As shown in Figure 3, four stray light eliminating apertures are placed between the front aperture and the rear aperture of the transfer radiometer, and the diameters of these four apertures are 21 mm, 20 mm, 19 mm, and 18 mm, in turn. In addition, the front and rear surfaces of all apertures and the inner wall of the radiance measuring tube are coated with black paint to maximize the suppression of stray light, which has an absorptivity up to more than 95% in the spectral range from 380 nm to 2500 nm. The simulation results show that the ability of the transfer radiometer to suppress stray light is better than 0.06%, which has little effect on the radiance measurement of the transfer radiometer.

In the on-orbit radiometric benchmark transfer chain, the transfer radiometer is required to measure the optical radiation from monochromatic laser-based and broad-spectrum incandescent lamp-based sources. The filter wheel has 12 mounting holes, 11 of which are used to mount narrowband interference filters with different center wavelengths, and the last hole is a through hole without any optical components. The transfer radiometer is divided into 11 filter channels and one filter-free channel by rotating the filter wheel by the motor. A 15-bit encoder is selected to feed back the filter wheel’s position to ensure that the position deviation of each rotation is better than 0.1^o^. The relevant parameters of 11 interference filters are shown in Table 2.

An interference filter’s central wavelength and spectral transmittance are affected by factors such as temperature and the angle of incidence of the ray. The spectral transmittance of the filter selected for the transfer radiometer is little affected by the temperature, and the change of the filter transmittance is better than 0.05% when the temperature changes 1 °C. During the operation of the transfer radiometer, the filters are placed in a temperature-controlled space where the temperature change is better than 0.5 °C, so the measurement error introduced by the temperature is minimal.

In a radiometric benchmark transfer chain, the transfer radiometer is used to directly measure laser optical power to calibrate its spectral power responsivity and to measure the spectral radiances of quasi-Lambertian sources, based on monochromatic lasers and incandescent lamps to calibrate the imaging spectrometer’s spectral radiance responsivity. The transmittance of the filter is greatly affected by the angle of incidence of the ray. The filter-free channel of the transfer radiometer is insensitive to the angle of incidence, so the transfer radiometer only performs spectral power responsivity calibration for the filter-free channel and completes the conversion of optical power responsivity to radiance responsivity. First, the laser output from a tunable laser source with a specific wavelength is introduced into an integrating sphere, and the transfer radiometer uses the filter-free channel and a filter channel to observe the integrating sphere source, instead of measuring the directly incident laser beam, to obtain the single wavelength transmittance of an interference filter. Then, by changing the output laser wavelength of the tunable laser, and repeating the above steps, the spectral transmittance curve of this interference filter can be obtained. After that, this filter channel’s spectral radiance responsivity curve can be derived from the filter-free channel’s spectral radiance responsivity curve and this filter’s spectral transmittance curve. Once the spectral radiance responsivity curves of the 11 filter channels of the transfer radiometer are obtained, accurate measurements of the spectral radiances of quasi-Lambertian sources can be achieved by using the transfer radiometer. The filter transmittance is measured in the same interference filter operating mode as when observing a quasi-Lambertian source by using the above method, which can effectively eliminate the effect of different angles of incidence of the optical radiation on the filter performance caused by different types of light sources. In addition, the interference filters used in the transfer radiometer have better out-of-band suppression capability than OD5 in the practical measurement spectral ranges of the detectors, reducing the effect of out-of-band light.

The transfer radiometer has a wide operating spectral range from 380 nm to 2350 nm. A silicon detector with a spectral response range of 190 nm to 1100 nm is used for the measurements in the visible to near-infrared spectral region. The detector that covers the short-wave infrared spectral region is a thermoelectrically cooled extended InGaAs detector operating over the spectral range of 0.8 µm to 2.4 µm. The radiance measuring tube is equipped and attached to a 21 mm diameter port on the integration sphere. The silicon and InGaAs detectors are respectively mounted in the two exit ports of the integrating sphere. The integrating sphere has a diameter of 70 mm and is coated with polytetrafluoroethylene (PTFE) that has a diffuse reflectance better than 95% in the spectral range of 380 nm to 2350 nm. As shown in Figure 3, two PTFE circular baffles are placed in the integrating sphere to prevent the first reflected light from entering the active areas of the detectors. The Si detector baffle has a diameter of 15 mm and the InGaAs detector baffle has a diameter of 14 mm.

In radiant power mode measurements, the front and rear apertures of the transfer radiometer are underfilled by laser beams, and in radiance mode measurements for quasi-Lambertian sources, the front and rear apertures of the transfer radiometer are overfilled by incident radiation.

**Figure 4:**
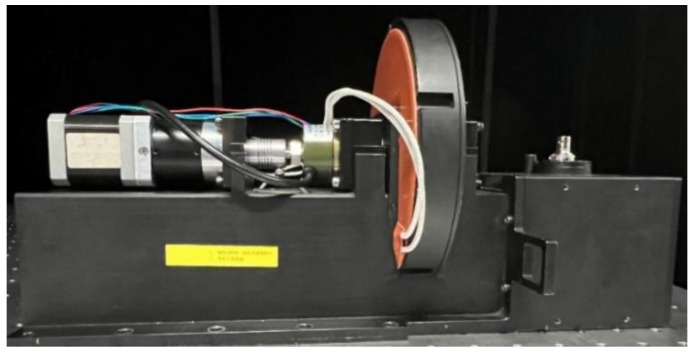
Picture of the transfer radiometer.

The Si detector is less affected by temperature, and the typical temperature variations of the environment in which the transfer radiometer is used are better than 1 °C. In the applied wavelength range, the photocurrent change of the Si detector of the transfer radiometer is better than 0.015% within a temperature change of 1 °C. Therefore, the effect of temperature on the Si detector is negligible. The operating temperature of the thermoelectrically cooled extended InGaAs detector is −85 °C. The housing of the filter wheel that filter combinations are mounted in can be temperature-controlled by using heating sheets. The temperature can be controlled to be slightly above room temperature, and the temperature control accuracy is better than 0.5 °C. This temperature adjustment ensures that the filters have stable spectral transmittances and are not affected by condensation. An actual picture of the transfer radiometer is shown in Figure 4.

## 3. Comparison Experiment

In order to verify the accuracy of the radiance responsivity of the transfer radiometer, the 852 nm filter channel of the transfer radiometer was selected, and the radiance measurement comparison experiment at a wavelength of 852.1 nm was carried out with a radiance meter developed by the National Institute of Metrology (NIM), China.

### 3.1. Power Responsivity Calibration

**Figure 5:**
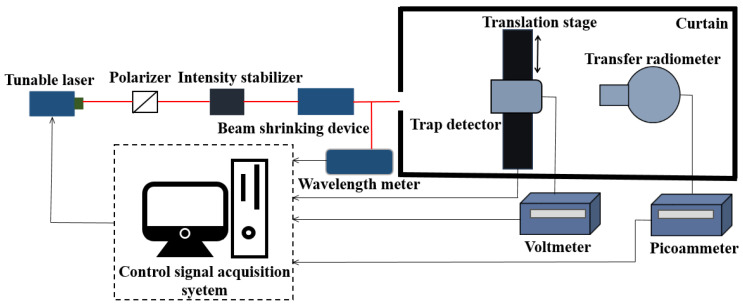
Schematic diagram of the experimental setup for the power responsivity calibration.

A trap detector was used as the transfer standard detector for the transfer radiometer’s power responsivity calibration, and the trap detector’s power responsivity was calibrated using a cryogenic radiometer, the primary radiometric standard for optical power measurement. The experimental setup for the power responsivity calibration of the transfer radiometer is shown in Figure 5. A tunable laser was used as the light source in this experiment, and the output laser wavelength of the tunable laser was measured by a laser wavelength meter. The output laser wavelength of the tunable laser was adjusted to 852.1 nm according to the feedback of the wavelength meter. This comparison experiment was performed to confirm the accuracy of the radiance responsivity of the transfer radiometer. The wavelength of the light source does not need to be strictly consistent with the central wavelength of the transfer radiometer narrowband filter, and it is only necessary to ensure that a wavelength of the light source used in the transfer radiometer power responsivity calibration experiment and the radiance measurement comparison experiment is the same. The laser beam passing through an intensity stabilizer was received by the trap detector placed in front of the transfer radiometer after shrinking the laser beam with a beam shrinking device. At first, the incident laser optical power was measured by the trap detector, and a voltmeter gave the output of the trap detector. Laser optical power was derived from trap detector power responsivity and voltmeter measurement values. After the measurement was completed, the trap detector was moved out of the optical path. The transfer radiometer was set to the filter-free channel by rotating the filter wheel, and was subsequently used to measure the incident laser optical radiation. The output current value of the silicon detector of the transfer radiometer was obtained with a picoammeter (Keithley 6485). The power responsivity of the transfer radiometer filter-free channel at a wavelength of 852.1 nm was calculated as the product of the current value measured by the picoammeter and the power value measured by the trap detector. After that, the radiance responsivity of the transfer radiometer filter-free channel at a wavelength of 852.1 nm was obtained from the conversion relationship of transfer radiometer power responsivity to radiance responsivity. The results are shown in Table 3.

### 3.2. Comparison Experiment of Radiance Measurement

**Figure 6:**
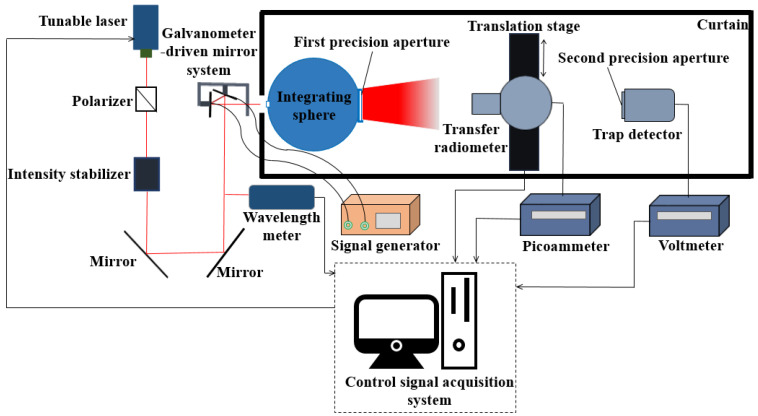
Schematic diagram of the experimental setup for the radiance measurement comparison.

The NIM radiance meter consists of a trap detector and two precision apertures. The spectral radiant power responsivity of the trap detector was directly traceable to a cryogenic radiometer. The first precision aperture of the radiance meter is located at the exit port of the integrating sphere, and the second precision aperture is located at the front of the trap detector. The power value measured by the trap detector and the geometry of the two aperture system yield the radiance value measured by the radiance meter, which is traceable to a cryogenic radiometer. The measurement uncertainty of the radiance meter is approximately 0.3% (k = 1). The experimental setup for the radiance measurement comparison of the transfer radiometer and NIM radiance meter is shown in Figure 6. The laser beam with a wavelength of 852.1 nm from a tunable laser passed through an intensity stabilizer, was reflected by two plane mirrors and a galvanometer-driven mirror system, and entered an integrating sphere with a diameter of 300 mm. The diameter of the first precision aperture of the radiance meter is 70.416 mm. There is a circular baffle with a diameter of 200 mm in the integrating sphere to ensure that the output light radiation of the integrating sphere source to be measured does not contain the first reflected light radiation of the incident light introduced into the integrating sphere. The function of galvanometer-driven mirror system is to suppress laser speckle effects. The transfer radiometer is placed between the two apertures of the radiance meter, and the front aperture of the transfer radiometer is approximately 275 mm away from the exit port of the integrating sphere. The optical axes of the transfer radiometer, the radiance meter, and the integrating sphere exit port were coincidentally after proper alignment. Following the method in the previous section, the transfer radiometer filter wheel was alternately turned to the filter-free channel and the 852 nm filter channel to measure the integrating sphere source. When observing an integrating sphere source with a wavelength of 852.1 nm, the transmittance of the narrowband interference filter with a center wavelength at 851.8 nm was obtained based on the Si detector output current values measured by the filter-free channel and the 852 nm filter channel of the transfer radiometer. The transmittance result is shown in Table 4. Based on this transmittance and the radiance responsivity of the filter-free channel at a wavelength of 852.1 nm, the radiance responsivity of the transfer radiometer 852 nm filter channel at a wavelength of 852.1 nm was calculated. Then, the radiance values of the integrating sphere source at a wavelength of 852.1 nm measured by the filter-free channel and the 852 nm filter channel of the transfer radiometer were obtained. After the measurement was completed, the transfer radiometer was removed to ensure that it would not block the field of view of the radiance meter. Next, the radiance value of the integrating sphere source was measured by the radiance meter. Table 5 shows the differences between the radiance values measured by the transfer radiometer filter-free channel and 852 nm filter channel, and the radiance value measured by the radiance meter at a wavelength of 852.1 nm.

### 3.3. Uncertainty Analysis

The measurement uncertainty sources in the comparison of radiance measurement are mainly divided into three categories: the radiance meter, the transfer radiometer, and the integrating sphere source used in the experiment. The measurement uncertainties of the radiance meter and the integrating sphere source are offered by the NIM. The present paper mainly focuses on the measurement uncertainty analysis of the transfer radiometer, especially the filter-free channel.

(1) Transfer Radiometer Power Responsivity Calibration Uncertainty

The transfer radiometer power responsivity calibration uncertainty is determined by the transfer radiometer measurement repeatability, the picoammeter measurement uncertainty, and the trap detector power responsivity calibration uncertainty. Transfer radiometer measurement repeatability is approximately 0.1% (k = 1). The measurement uncertainty of the picoammeter is approximately 0.05% (k = 1). The power responsivity of the trap detector is calibrated by a cryogenic radiometer, and the calibration uncertainty is approximately 0.05% (k = 1).

(2) Transfer Radiometer Power Responsivity to Radiance Responsivity Conversion Measurement Uncertainty

From Equation (10), it can be seen that the conversion from power responsivity of the transfer radiometer to radiance responsivity is mainly affected by the measurement uncertainty of the front aperture diameter D, the rear aperture diameter d and the distance l between the two apertures. A universal tool microscope was used to measure the diameters of the front and rear apertures. The measurement uncertainties of diameters of the front and rear apertures were about 0.04% (k = 1) and 0.08% (k = 1), respectively. The distance between the front and rear apertures was measured by a coordinate measuring machine and the measurement uncertainty of the distance between two apertures was about 0.04% (k = 1). The installation deviation between the front and rear apertures of the transfer radiometer will also affect the power responsivity to radiance responsivity conversion uncertainty. The eccentricity of the two apertures of the transfer radiometer is about 0.2 mm, and the parallelism of the two apertures is about 5′. The measurement uncertainty introduced by eccentricity is about 0.0001% (k = 1), and the measurement uncertainty introduced by tilt is about 0.0002% (k = 1). The combined uncertainty for converting transfer radiometer power responsivity into radiance responsivity is about 0.098% (k = 1).

(3) Transfer Radiometer Radiance Measurement Uncertainty

The radiance measurement uncertainty of the transfer radiometer is mainly affected by the transfer radiometer measurement repeatability and the picoammeter measurement uncertainty. The measurement repeatability of the transfer radiometer is about 0.1% (k = 1), and the measurement uncertainty of the picoammeter is about 0.05% (k = 1).

(4) Detector Linearity and Stray Light Effects

In the transfer radiometer optical power responsivity calibration experiment and the radiance measurement comparison experiment, the measured signals obtained with the Si detector differed by two orders of magnitude. However, the Si detector has good linearity, and the measurement uncertainty introduced by the nonlinear responsivity of the Si detector is better than 0.1% (k = 1). Referring to the simulation result, the transfer radiometer measurement uncertainty introduced by stray light is about 0.06% (k = 1).

(5) Temperature Effects

During the experiment, the temperature in the laboratory was maintained at 23 ± 0.2 °C, and the effect of temperature change on the filter’s transmittance and the Si detector’s spectral responsivity could be ignored.

Table 6 shows the measurement uncertainty estimation for the radiance measurement comparison experiment of the transfer radiometer filter-free channel. The transfer radiometer filter channel radiance measurement uncertainty is combined with the filter-free channel radiance measurement uncertainty and the filter transmittance measurement uncertainty. The single wavelength transmittance of the transmission radiometer filter was obtained in the radiance measurement comparison experiment, and the measurement uncertainty of single wavelength transmittance was about 0.02% (k = 1). Therefore, the combined uncertainty for the radiance measurement comparison experiment of the transfer radiometer filter channel is about 0.43% (k = 1). Furthermore, the relative deviations of the radiances measured by the transfer radiometer filter-free channel and the 852 nm filter channel, and the radiance measured by the radiance meter are better than 0.36%, which is better than the combined uncertainty for the radiance measurement comparison experiment. Accordingly, we can assume that the transfer radiometer can achieve a radiance measurement uncertainty better than 0.3% (k = 1).

## 4. Conclusions

A transfer radiometer with power responsivity to radiance responsivity conversion uncertainty better than 0.1% (k = 1) and radiance measurement uncertainty better than 0.3% (k = 1) has been developed for a radiometric benchmark transfer chain. The radiance measurement comparison experiment was conducted between the transfer radiometer and the NIM radiance meter to verify the radiance measurement accuracy of the transfer radiometer. The same integrating sphere source was measured by the transfer radiometer and the radiance meter, and the non-uniformity was about 0.2% (k = 1) within the observed area of the integrating sphere source. The measurement uncertainty of the radiance meter is 0.3% (k = 1), the measurement uncertainty of the transfer radiometer is about 0.23% (k = 1), and the combined uncertainty of the radiance measurement comparison experiment is about 0.43% (k = 1). The relative deviation of radiance measurements between the transfer radiometer and the radiance meter is better than 0.36%, which is better than the combined uncertainty of the radiance measurement comparison experiment. It has been verified that the transfer radiometer can complete the conversion from power responsivity to radiance responsivity with an uncertainty better than 0.1% (k = 1) and achieves the radiance measurement uncertainty goal better than 0.3% (k = 1). The developed transfer radiometer is expected to be used as a reference instrument whose spectral power responsivity is directly traceable to a cryogenic radiometer and can be converted into spectral radiance responsivity for radiometric calibrations of remote sensing instruments such as imaging spectrometers. This is of great interest for improving the radiometric calibration accuracy of on-orbit remote sensing instruments.

## Figures and Tables

**Table 1:** Viewing angles of the transfer radiometer.

Full radiance-measurement angle β	8.429°
Nominal viewing angle θ	4.788°
Unvignetted FOV α	1.137°
Equivalent FOV $\theta_{m}$	4.786°

**Table 2:** Design parameters of 11 filters.

Channel	Diameter (mm)	Center Wavelength (nm)	Bandwidth (nm)
1	25.4	404.1	10.6
2	25.4	532.2	10.6
3	25.4	632.8	9.5
4	25.4	780.4	9.8
5	25.4	851.8	9.9
6	25.4	940.4	10.2
7	25.4	1030.2	11.0
8	25.4	1239.7	10.5
9	25.4	1308.6	8.7
10	25.4	1547.5	9.8
11	25.4	2051.4	14.8

**Table 3:** The experimental results of the power responsivity calibration of the filter-free channel of the transfer radiometer.

Wavelength (nm)	Laser Power (mW)	Power Responsivity of the Transfer Radiometer (A/W)	Radiance Responsivity of the Transfer Radiometer (10^−8^ A/(W/m^2^/sr))
852.1	0.8326	0.0362	3.9729

**Table 4:** The transmittance of the narrowband interference filter for 852 nm filter channel at a wavelength of 852.1 nm.

Center Wavelength of the Filter (nm)	Laser Wavelength (nm)	Transmittance
851.8	852.1	0.981

**Table 5:** Relative deviations of measurements from the transfer radiometer and the radiance meter at a wavelength of 852.1 nm.

Channel	Radiance Measured by the Transfer Radiometer A/(W/m^2^/sr)	Radiance Measured by the Radiance Meter A/(W/m^2^/sr)	Relative Deviation (%)
Filter-free channel	6.160	6.140	0.33
852 nm filter channel	6.162	6.140	0.36

**Table 6:** Uncertainty for the radiance measurement comparison experiment of the transfer radiometer filter-free channel.

Source of Uncertainty	Uncertainty Contribution (%) (k = 1)
NIM radiance meter	0.3
Integrating sphere source uniformity	0.2
Transfer radiometer	0.23
Power measurement repeatability	0.1
Picoammeter reading in power responsivity calibration	0.05
Trap detector	0.05
Front aperture diameter	0.04
Rear aperture diameter	0.08
Distance between the front and rear apertures	0.04
Eccentricity of the front and rear apertures	0.0001
Parallelism of the front and rear apertures	0.0002
Radiance measurement repeatability	0.1
Picoammeter reading in radiance comparison	0.05
Detector response linearity	0.1
Stray light	0.06
Combined uncertainty	0.43
